# The Effects of Rice Bran on Neuroinflammation and Gut Microbiota in Ovariectomized Mice Fed a Drink with Fructose

**DOI:** 10.3390/nu16172980

**Published:** 2024-09-04

**Authors:** Yu-Wen Chao, Yu-Tang Tung, Suh-Ching Yang, Hitoshi Shirakawa, Li-Han Su, Pei-Yu Loe, Wan-Chun Chiu

**Affiliations:** 1 School of Nutrition and Health Sciences, College of Nutrition, Taipei Medical University, Taipei 110, Taiwan; yuwenchao07@gmail.com (Y.-W.C.); sokei@tmu.edu.tw (S.-C.Y.); da07110005@tmu.edu.tw (L.-H.S.); ma07111019@tmu.edu.tw (P.-Y.L.); 2 Graduate Institute of Biotechnology, National Chung Hsing University, Taichung 402, Taiwan; peggytung@nchu.edu.tw; 3 Advanced Plant and Food Crop Biotechnology Center, National Chung Hsing University, Taichung 402, Taiwan; 4 Cell Physiology and Molecular Image Research Center, Wan Fang Hospital, Taipei Medical University, Taipei 116, Taiwan; 5 Research Center of Geriatric Nutrition, College of Nutrition, Taipei Medical University, Taipei 110, Taiwan; 6 International Education and Research Center for Food and Agricultural Immunology, Graduate School of Agricultural Science, Tohoku University, Sendai 980-8572, Japan; shirakah@tohoku.ac.jp; 7 Department of Nutrition, Wan Fang Hospital, Taipei Medical University, Taipei 116, Taiwan

**Keywords:** rice bran, tea seed oil, neuroinflammation, gut microbiota, fructose drink, menopause, short-chain fatty acid

## Abstract

Rice bran, which is abundant in dietary fiber and phytochemicals, provides multiple health benefits. Nonetheless, its effects on neuroinflammation and gut microbiota in postmenopausal conditions are still not well understood. This study investigated the effects of rice bran and/or tea seed oil supplementation in d-galactose-injected ovariectomized (OVX) old mice fed a fructose drink. The combination of d-galactose injection, ovariectomy, and fructose drink administration creates a comprehensive model that simulates aging in females under multiple metabolic stressors, including oxidative stress, estrogen deficiency, and high-sugar diets, and allows the study of their combined impact on metabolic disorders and related diseases. Eight-week-old and 6–8-month-old female C57BL/6 mice were used. The mice were divided into six groups: a sham + young mice, a sham + old mice, an OVX + soybean oil, an OVX + soybean oil with rice bran, an OVX + tea seed oil (TO), and an OVX + TO with rice bran diet group. The OVX groups were subcutaneously injected with d-galactose (100 mg/kg/day) and received a 15% (*v*/*v*) fructose drink. The rice bran and tea seed oil supplementation formed 10% of the diet (*w*/*w*). The results showed that the rice bran with TO diet increased the number of short-chain fatty acid (SCFA)-producing *Clostridia* and reduced the number of endotoxin-producing *Tannerellaceae*, which mitigated imbalances in the gut–liver–brain axis. Rice bran supplementation reduced the relative weight of the liver, levels of hepatic triglycerides and total cholesterol; aspartate transaminase and alanine aminotransferase activity; brain levels of proinflammatory cytokines, including interleukin-1β and tumor necrosis factor-α; and plasma 8-hydroxy-2-deoxyguanosine. This study concludes that rice bran inhibits hepatic fat accumulation, which mitigates peripheral metaflammation and oxidative damage and reduces neuroinflammation in the brain.

## 1. Introduction

The incidences of neurodegenerative diseases and metabolic syndrome are higher in women after menopause [1,2,3]. The changes in sex hormones that occur during menopause, including the decrease in the amount of estrogen produced by the ovaries, impairs the utilization of glucose in the brain and triggers neuroinflammation, leading to cognitive dysfunction [4,5,6]. The effects of estrogen in skeletal muscle, liver, adipose tissue, and immune cells are associated with insulin sensitivity and the prevention of lipid accumulation and inflammation [7]. An increasing number of studies have attempted to slow the progression of neurodegenerative diseases and metabolic syndrome by altering the composition of the gut microbiota and their metabolites, such as short-chain fatty acids (SCFAs) [8,9]. When low-grade inflammation is induced by metabolic diseases, which often occurs in women after menopause and is known as metaflammation, the dominant gut microbiota change, with the changes that occur being influenced by the specific disease, and intestinal permeability increases, enabling harmful bacteria and toxins from the lumen to translocate into the bloodstream more easily [10,11,12]. Once these toxins enter the bloodstream, they bind to cluster of differentiation 14 (CD14) on macrophages and monocytes and are recognized by toll-like receptor 4 (TLR4), which exacerbates metaflammation and promotes liver damage, leading to a vicious cycle of hyperglycemia and dyslipidemia [13,14,15,16]. Additionally, the free fatty acids and cytokines released because of abnormal blood sugar levels and a fatty liver cross the blood–brain barrier and trigger immune responses by the microglia and astrocytes in the brain, causing the chronic secretion of reactive oxidative species, NO, and proinflammatory cytokines, which further induce neural insulin resistance and synaptic loss and contribute to the impairment of cognition [15,17,18].

The Mediterranean–Dietary Approaches to Stop Hypertension (DASH) Intervention for Neurodegenerative Delay (MIND) diet was proposed in 2015. A high level of adherence to this diet among patients with an average age of 81.4 years was reported to have delayed the worsening of global cognitive function by 7.5 years [19]. The MIND diet score was also reported to be inversely associated with high-density lipoprotein levels and the risk of general obesity in Iranian adults [20]. A review of the association between dietary patterns and dyslipidemia also revealed that consumption of a DASH diet was associated with a lower risk of metabolic dyslipidemia [21]. Olive oil and whole grains are two of the ten categories of foods emphasized in the MIND. Olive oil and whole grain consumption hinders the progression of neurodegenerative diseases and metabolic syndrome [22,23,24]. Similarly to olive oil, tea seed oil is abundant in monounsaturated fatty acids (MUFAs) [25]. In an AlCl_3_-induced Alzheimer disease rat model, treatment with tea seed oil (1.5 or 3 mg/kg/day) through oral gavage for 8 weeks significantly improved cognitive performance in the Morris water maze by inhibiting the production of malondialdehyde, interleukin (IL)-1β, IL-6, and tumor necrosis factor (TNF)-α. Additionally, the dominant gut microbiota shifted from *Enterobacteriaceae* to *Lactobacillus* after the tea seed oil intervention [26]. Our previous study demonstrated that tea seed oil consumption prevented obesity in ovariectomized (OVX) mice with high-fat diet (HFD)-induced obesity [27]. In addition, tea seed oil was discovered to relieve the effects of metabolic disorders and oxidative stress in rats fed an HFD or high-fructose diet [28]. Similarly to whole grains, rice bran contains numerous phytochemicals [29]. Among them, γ-oryzanol is most often used to ameliorate neuroinflammation and fat accumulation in the liver [30,31]. To date, no study on the effects of tea seed oil and rice bran on multifactorial menopausal disorders exists. Therefore, the present study investigated the effects of rice bran and tea seed oil supplementation in old d-galactose-injected OVX mice fed a fructose drink for 8 weeks.

## 2. Materials and Methods

### 2.1. Animals

Female C57BL/6 mice aged 8 weeks or 6–8 months were purchased from BioLASCO (Yilan, Taiwan). The mice were housed in a temperature- (22 ± 2 °C) and humidity-controlled (50 ± 10%) environment with a 12 h light/dark cycle and had free access to food and water. The experimental procedure was approved by the Institutional Animal Care and Use Committee of Taipei Medical University, Taipei, Taiwan (LAC-2020-0161).

### 2.2. Experimental Design

Eight mice aged 8 weeks and 8 mice aged 6–8 months underwent a sham operation, and 32 mice aged 6–8 months were OVX. After 3 weeks of recovery from surgery, the animals were divided into the following six groups: sham + young mice (SY), sham + old mice (SO), OVX + soybean oil diet (OS), OVX + soybean oil with rice bran diet (OSR), OVX + tea seed oil diet (OT), and OVX + tea seed oil with rice bran diet (OTR). When the intervention commenced, the mice were fed a modified AIN-93M diet that included soybean oil, soybean oil with rice bran, tea seed oil, or tea seed oil with rice bran for 8 weeks. The mice in the OVX groups were injected subcutaneously with d-galactose (100 mg/kg body weight; Sigma-Aldrich, St. Louis, MO, USA) and provided with a 15% (*v*/*v*) fructose drink for 8 weeks. Each group’s diet was a modified version of the AIN-93M diet with 10% (*w*/*w*) rice bran and/or tea seed oil added in the intervention groups, with the proportion of added bran or oil determined on the basis of other studies [32,33]. Rice bran is obtained from a blend of Koshihikari and Hitomebore semi-defatted rice bran from Japan. The rice bran contained 22% carbohydrates, 18.5% protein, 9.7% fat, and 34.6% dietary fiber. The active compounds in rice bran included total polyphenols (5.5 mg/g), ferulic acid (115 μg/g), and γ-oryzanol (119.1 μg/g). The soybean and tea seed (from *Camellia Oleifera*) oils were produced by Taiwan Sugar Corporation and purchased from the supermarket. The composition of the diets is presented in Supplementary Table S1.

### 2.3. Biochemical Assay and Enzyme-Linked Immunosorbent Assay

At the end of the experiment, the mice were anesthetized by the intraperitoneal injection of 40 mg/kg Zoletil (Virbac, Carros, France) plus 20 mg/kg Rompun (Bayer, Leverkusen, Germany) and sacrificed using cardiac puncture. Their brains, plasma, and livers were collected and stored at −80 °C until analysis. Plasma glucose, triglyceride (TG), total cholesterol (TC), aspartate transaminase (AST), and alanine aminotransferase (ALT) levels were determined using an automated biochemistry analyzer. The insulin and oxidative damage marker (8-hydroxy-2-deoxyguanosine (8-OHdG)) levels in the plasma, liver lipid (TG and TC) levels, and proinflammatory cytokine (IL-1β, IL-6, and TNF-α) levels in the frontal cortex of the brain were measured using a commercial enzyme-linked immunosorbent assay kit (insulin: Cat. No. 10-1249-01, Mercodia, Winston Salem, NC, USA; 8-OHdG: Lot No. D61YXN2J1T, Elabscience Biotechnology, Houston, TX, USA; TG: Lot No. 448536, Randox Laboratories, County Antrim, UK; TC: Lot No. 497567, Randox Laboratories, County Antrim, UK; IL-1β: Lot No. 8M9PKY4UB8, Elabscience Biotechnology, TX, USA; IL-6: Lot No. 8PZ7G6GLQ4, Elabscience Biotechnology, TX, USA; TNF-α: Lot No. QH1HWHCV1M, Elabscience Biotechnology, TX, USA). Insulin resistance was calculated using the homeostatic model assessment for insulin resistance (HOMA-IR) according to the following formula: glucose (mg/dL) × insulin (µg/L)]/405.

### 2.4. Morris Water Maze

The Morris water maze task was performed as previously described [34]. The setup included a circular pool (100 cm in diameter) filled with water that was maintained at 22 ± 2 °C. A transparent platform was positioned in the southwest quadrant of the pool and submerged 0.5–1 cm below the water surface. During the acquisition trial of the task, the mice were trained to locate the platform for 5 days and completed four trials per day, with each lasting 60 s. The time required for the mice to locate the platform was recorded as their escape latency. The mice that could not locate the platform within 60 s were placed by the experimenter on the platform, where they stayed for 15 s. On the sixth day, the platform was removed, and a probe trial was conducted. Each mouse was allowed to explore for 30 s before several parameters, including the time spent in and the path within the target quadrant, were measured.

### 2.5. 16S rRNA Sequencing

Extracted cecal DNA was subjected to amplification, which was completed using a universal V3–V4 primer and a thermocycler (Applied Biosystems 2720, Thermo Fisher Scientific, Vacaville, CA, USA) followed by PCR purification (Agencourt AMPure XP, Beckman Coulter, Illumina, San Diego, CA, USA). Microbiota were classified with reference to the SILVA v132 taxonomy database (https://www.arb-silva.de, accessed on 30 September 2022) and by using the DADA2 workflow (https://github.com/benjjneb/dada2, accessed on 30 September 2022). Differential features were identified using the linear discriminant analysis (LDA) effect size (LDA values of >3.0 were used as thresholds).

### 2.6. Short-Chain Fatty Acid Analysis

Fecal samples were suspended in 1 mL of water with 0.5% phosphoric acid (CAS No.: 7664-38-2, Honeywell Specialty Chemicals Seelze, Seelze, Germany) and extracted using ethyl acetate (CAS No.: 141-78-6, Macron Fine Chemicals, Radnor, PA, USA). The organic phase was analyzed using an apparatus comprising a gas chromatograph (7820A GC, Agilent, Santa Clara, CA, USA), a column (Nukol Capillary Column, Supelco, Bellefonte, PA, USA), a mass spectrometer (5977B EI MSD, Agilent, Santa Clara, CA, USA) and the Agilent MassHunter Workstation software version 10.0.368 (Agilent, Santa Clara, CA, USA). We injected 1 µL of the standard (Volatile Free Acid Mix, Lot No. LRAC0113, Sigma-Aldrich, St. Louis, MO, USA) or sample into the gas chromatograph, which was separated using nitrogen as the mobile phase. The separated substances were then broken into ionized fragments and passed through a detector for recognition based on their mass-to-charge ratios (*m*/*z* ratios). Short-chain fatty acids were identified and calculated on the basis of the retention times of the standard.

### 2.7. Statistical Analysis

Statistical analysis was performed using GraphPad Prism (Version 9, GraphPad Software, San Diego, CA, USA), and the data are presented as means ± standard errors of the mean. A *t*-test was used to compare the SO or OS group with the SY group, and one-way analysis of variance followed by Tukey’s test was used to identify significant differences between the OVX groups. Correlations of the brain and liver biomarkers with specific microbiota in the gut were analyzed using Pearson’s correlation. A *p* value of <0.05 indicated significance.

## 3. Results

### 3.1. Effects of Rice Bran and Tea Seed Oil Supplementation on Body and Uterine Weight in Ovariectomized Old Mice Fed a Fructose Drink

**Figure 1:**
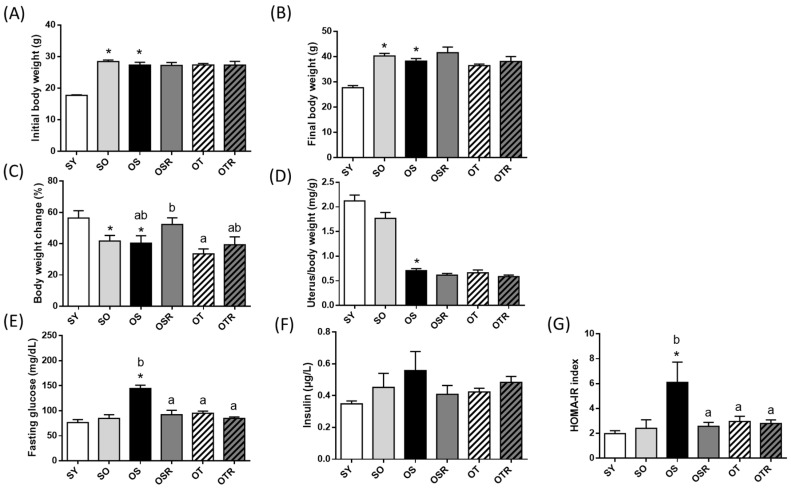
Effects of rice bran and tea seed oil supplementation on body and uterine weight. (**A**) Initial body weight, (**B**) final body weight, (**C**) body weight change during the experiment, (**D**) relative uterine weight, (**E**) fasting glucose, (**F**) insulin, and (**G**) HOMA-IR index. HOMA-IR: homeostatic model assessment for insulin resistance; SY: sham + young mice; SO: sham + old mice; OS: OVX mice + soybean oil diet; OSR: OVX mice + soybean oil with rice bran diet; OT: OVX mice + tea seed oil diet; OTR: OVX + tea seed oil with rice bran diet; OVX: ovariectomized. Values are presented as the mean ± SEM (*n* = 8). Asterisks (*) indicate significant differences between the SO or OS group compared with the SY group. Different letters indicate significant differences between the OVX groups.

With the exception of those in the SY group, the mice had similar body weights at baseline (Figure 1A). After d-galactose and the fructose drink treatment was administered for 8 weeks, the final average body weights in the OVX groups did not differ significantly (Figure 1B). However, the OSR mice exhibited greater proportional weight gain (52.3 ± 4.3%) than did the OT mice (33.5 ± 3.2%) at the end of the study (Figure 1C). As presented in Figure 1D, the relative uterine weight of the OVX groups decreased significantly, indicating that the bilateral removal of the ovaries was successful. Compared with those in the SY groups, the mice in the OVX groups had a lower relative uterine weight.

### 3.2. Effects of Rice Bran and Tea Seed Oil Supplementation on Glucose Utilization and Insulin Resistance in Ovariectomized Old Mice Fed a Fructose Drink

The fasting glucose levels in the OS group (144.4 ± 6.8 mg/dL) were higher than those in the two sham operation groups (76.3 ± 6.0 mg/dL for the SY group and 84.7 ± 7.5 mg/dL for the SO group). Supplementation with rice bran, tea seed oil, or both was associated with reductions in fasting glucose levels of 36.1%, 34.2%, and 41.3%, respectively (Figure 1E). However, insulin levels did not differ significantly between the groups (Figure 1F). The HOMA-IR index is generally used to determine the presence of insulin resistance [35]. The HOMA-IR index was significantly greater in the OS group (6.1 ± 1.6) than in the SY group (2.0 ± 0.2). Among the OVX groups, the OSR and OTR groups had lower HOMA-IR index values, indicating they had lower insulin resistance (2.6 ± 0.3 and 2.8 ± 0.3, respectively; Figure 1G).

### 3.3. Effects of Rice Bran and Tea Seed Oil Supplementation on Lipid Accumulation in Ovariectomized Old Mice Fed a Fructose Drink

**Figure 2:**
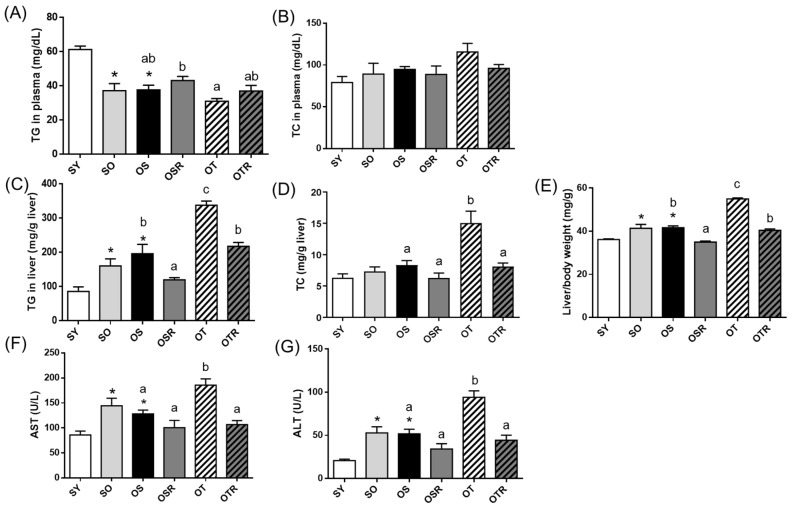
Effects of rice bran and tea seed oil supplementation on lipid levels and liver function. Plasma (**A**) TG and (**B**) TC and hepatic (**C**) TG and (**D**) TC levels; (**E**) relative liver weight; and (**F**) AST and (**G**) ALT activities. SY: sham + young mice; SO: sham + old mice; OS: OVX mice + soybean oil diet; OSR: OVX mice + soybean oil with rice bran diet; OT: OVX mice + tea seed oil diet; OTR: OVX mice + tea seed oil with rice bran diet; TG: triglyceride; TC: total cholesterol; AST: aspartate transaminase; ALT: alanine transaminase; OVX: ovariectomized. Values are presented as the mean ± SEM (*n* = 8). Asterisks (*) indicate significant differences between the SO or OS group compared with the SY group. Different letters indicate significant differences between the OVX groups.

Compared with the SY mice, the SO and OS groups had significantly lower plasma TG levels and significantly higher liver TG levels (Figure 2A,C). The liver TG levels were higher in the OT group than in the OS group (Figure 2C). The rice-bran-fed mice in the OSR and OTR groups had lower liver TG levels and lower relative liver weights than did those in the OS and OT groups, respectively (Figure 2C,E). When tea seed oil rather than soybean oil was administered for 8 weeks, hepatic TC level and AST and ALT activities were significantly higher in the groups fed tea seed oil than in those fed soybean oil (Figure 2D,F,G). Compared with the OT group, the OTR group had significantly lower levels of hepatic TG and TC and lower AST and ALT activity (Figure 2C,D,F,G).

### 3.4. Effects of Rice Bran and Tea Seed Oil Supplementation on Peripheral Oxidative Damage and Neuroinflammation in Ovariectomized Old Mice Fed a Fructose Drink

**Figure 3:**
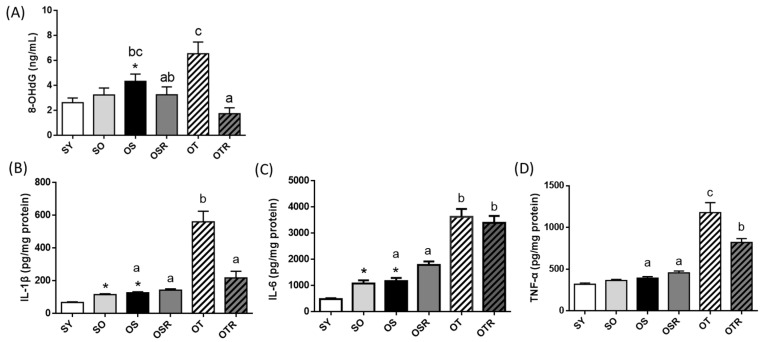
Effects of rice bran and tea seed oil supplementation on the (**A**) concentration of 8-OHdG in plasma and the levels of (**B**) IL-1β, (**C**) IL-6, and (**D**) TNF-α in the frontal cortex of the brain. SY: sham + young mice; SO: sham + old mice; OS: OVX mice + soybean oil diet; OSR: OVX mice + soybean oil with rice bran diet; OT: OVX mice + tea seed oil diet; OTR: OVX mice + tea seed oil with rice bran diet; 8-OHdG: 8-hydroxy-2-deoxyguanosine; OVX: ovariectomized; IL: interleukin; TNF-α: tumor necrosis factor-α. Values are presented as the mean ± SEM (*n* = 6–7). Asterisks (*) indicate significant differences between the SO or OS group compared with the SY group. Different letters indicate significant differences between the OVX groups.

Peripheral oxidative damage was evaluated by measuring the levels of DNA oxidation products. The level of 8-OHdG was higher in the OS group (4.3 ± 0.6 ng/mL) than in the SY group (2.6 ± 0.4 ng/mL). Compared with the OT groups (6.5 ± 0.9 ng/mL), the OTR groups exhibited significantly lower 8-OHdG production (1.7 ± 0.5 ng/mL; Figure 3A). Compared with the SY group, the SO and OS groups had higher levels of brain IL-1β, IL-6, and TNF-α (SO: 1.7, 2.2, and 1.1 times higher, respectively; OS: 1.9, 2.4, and 1.2 times higher, respectively; Figure 3B–D). When tea seed oil rather than soybean oil was administered for 8 weeks, the levels of IL-1β, IL-6, and TNF-α in the OT group were 441%, 310%, and 299% higher, respectively, than those in the OS group (Figure 3B–D). The OTR group had lower levels of brain IL-1β and TNF-α than did the OT group (Figure 3B,D).

### 3.5. Effects of Rice Bran and Tea Seed Oil Supplementation on Cognition in Ovariectomized Old Mice Fed a Fructose Drink

**Figure 4:**
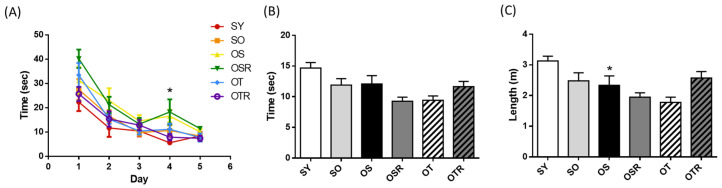
Effects of rice bran and tea seed oil supplementation on cognitive performance in the Morris water maze. (**A**) Escape latency during the acquisition trial and the (**B**) time spent and (**C**) path length in the target quadrant during the probe trial after the platform removal. SY: sham + young mice; SO: sham + old mice; OS: OVX mice + soybean oil diet; OSR: OVX mice + soybean oil with rice bran diet; OT: OVX mice + tea seed oil diet; OTR: OVX mice + tea seed oil with rice bran diet; OVX: ovariectomized. Values are presented as the mean ± SEM (*n* =6–7). Asterisks (*) indicate significant differences between the SO or OS group compared with the SY group.

In the Morris water maze acquisition trial, OS mice took longer to find the platform on day 4 (16.6 ± 2.6 s) compared with SY mice (5.6 ± 0.4 s). The escape latency during the acquisition trial did not differ significantly among the different OVX groups (Figure 4A). Mice aged 6–8 months may already be at an age where it is difficult to induce noticeable aging effects; this may explain why there was no significant difference in cognitive function compared with age-matched mice that did not undergo OVX and d-galactose treatment. During the probe trial, the time spent and the path lengths in the target quadrant did not differ significantly among the OVX groups (Figure 4B,C). There are two possible explanations. First, the natural aging process may have already triggered age-related changes in these mice, making it more difficult to enhance cognitive function through interventions. Second, the dosage used in this study might be insufficient to improve cognitive function in this multi-factor-induced animal model.

### 3.6. Effects of Rice Bran and Tea Seed Oil Supplementation on Gut Microbial Composition and Metabolites in Ovariectomized Old Mice Fed a Fructose Drink

**Figure 5:**
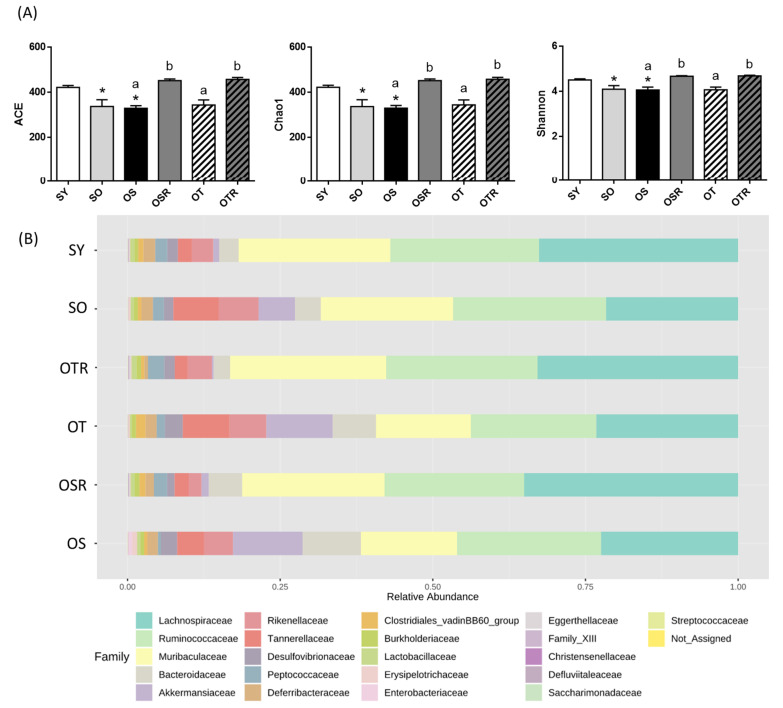

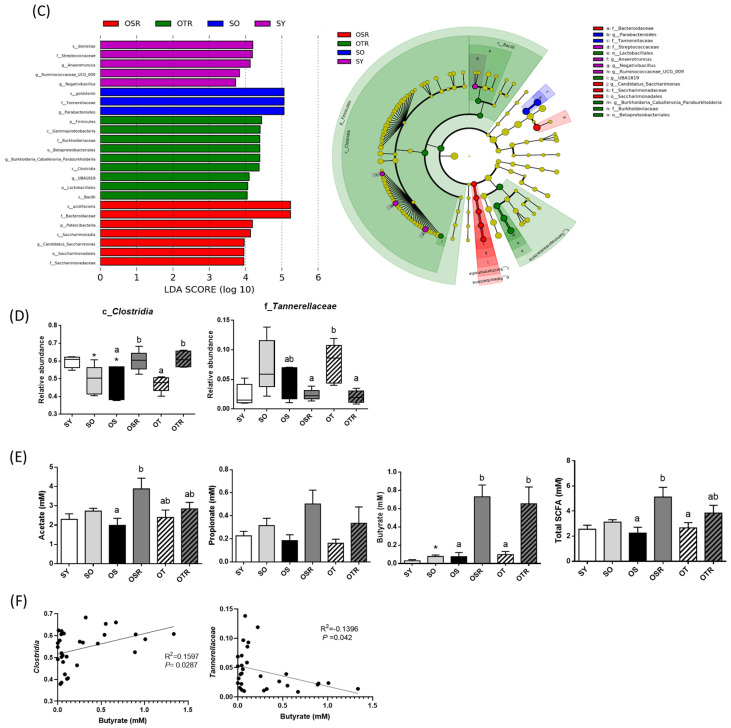
Effects of rice bran and tea seed oil supplementation on gut microbial composition and short-chain fatty acid levels. (**A**) Alpha diversity of the gut microbiota. (**B**) The relative abundance of gut microbiota at the family level. (**C**) Greatest differences in the gut microbiota between the different groups. Only taxa with a significant LDA threshold value of >3 are shown. (**D**) Relative abundance of *c_Clostridia* and *f_Tannerellaceae*. (**E**) Short-chain fatty acids (acetate, propionate, and butyrate). (**F**) The correlation between butyrate and *Clostridia* and *Tannerellaceae*. LDA: linear discriminant analysis; SY: sham + young mice; SO: sham + old mice; OS: OVX mice + soybean oil diet; OSR: OVX mice + soybean oil with rice bran diet; OT: OVX mice + tea seed oil diet; OTR: OVX mice + tea seed oil with rice bran diet. Values are presented as the mean ± SEM (*n* = 5). Asterisks (*) indicate significant differences between the SO or OS group compared with the SY group. Different letters indicate significant differences between the OVX groups.

We analyzed the gut microbiota composition of the mice. The alpha diversity index values of richness (ACE and Chao1) and richness and evenness (Shannon) were significantly lower in the SO and OS groups than in the SY group. The ACE, Chao1, and Shannon index values were significantly higher in the OSR and OTR groups than in the OS and OT groups, respectively (Figure 5A). The differences in the gut microbiota among the groups were analyzed. Regarding the relative abundance of the gut microbiome at the family level (Figure 5B), in the SO and OS groups, the relative abundance of *Clostridia* was lower than that in the SY group. The OSR and OTR groups exhibited a significantly higher relative abundance of Clostridia (60.0 ± 2.5% and 61.1 ± 2.0%, respectively) than did the OS and OT groups (47.8 ± 4.1% and 47.2 ± 1.9%, respectively; Figure 5D). Moreover, the OSR and OTR groups exhibited a lower relative abundance of *Tannerellaceae* than did the OS and OT groups, respectively (Figure 5C,D).

The primary metabolites that the intestinal microbiota ferment are SCFAs, mainly acetate, propionate, and butyrate [36]. Regarding the differences among the OVX groups, the OSR group had higher fecal acetate levels (3.9 ± 0.6 mM) than did the OS group (2.0 ± 0.4 mM; Figure 5E). Butyrate levels were higher in the OSR and OTR groups (0.73 ± 0.13 and 0.65 ± 0.18 mM, respectively) than in the OS and OT groups (0.08 ± 0.04 and 0.10 ± 0.03 mM, respectively; Figure 5E). Total SCFA levels were higher in the OSR and OTR groups than in the OS and OT groups, respectively (Figure 5E). The correlation between SCFAs and the gut microbiomes showed that butyrate levels were positively correlated with Clostridia (R^2^ = 0.1597; *p* = 0.0287) but negatively correlated with *Tannerellaceae* (R^2^ = −0.1396; *p* = 0.042).

### 3.7. Effects of Rice Bran and Tea Seed Oil Supplementation on the Gut–Liver–Brain Axis in Ovariectomized Old Mice Fed a Fructose Drink

**Figure 6:**
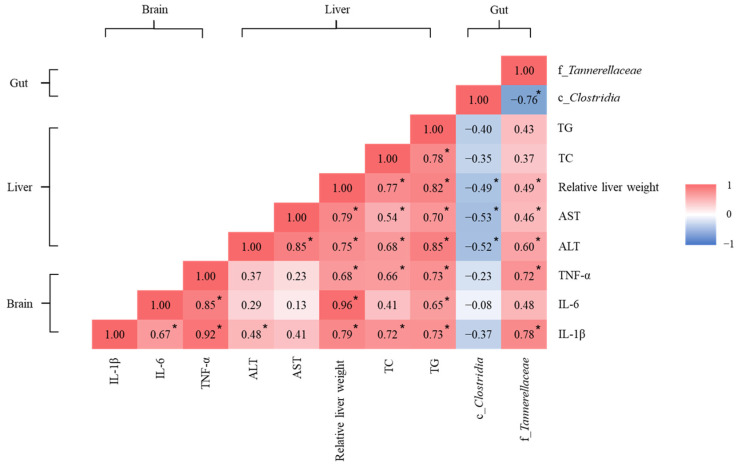
Heatmap of linear regression for biomarkers of the brain and liver with specific gut microbiota. IL: interleukin; TNF-α: tumor necrosis factor-α; ALT: alanine transaminase; AST: aspartate transaminase; TC: total cholesterol; TG: triglyceride. Colors were assigned according to the distribution of the Pearson correlation coefficient: red and blue represent positive and negative correlations, respectively. * *p* < 0.05, significant correlations.

Linear regression was performed to explore the correlations of brain and liver biomarkers with specific gut microbiota (Figure 6). Tannerellaceae abundance (greater in mice fed with tea seed oil) was significantly positively correlated with brain IL-1β and TNF-α levels, relative liver weight, and plasma liver function biomarker (AST and ALT) levels; Clostridia abundance (greater in mice fed with rice bran) was significantly negatively correlated with relative liver weight and liver AST and ALT levels. Levels of IL-1β and TNF-α in the brain significantly positively correlated with relative liver weight and hepatic TG and TC contents.

## 4. Discussion

Women who have undergone menopause exhibit higher rates of neurodegenerative diseases and metabolic syndrome than those who have not [1,2,3]. Because of the burden of these diseases on caregivers and the health-care system, dietary modification for such individuals has become increasingly crucial. Evidence links the MIND diet to beneficial effects on cognition and metabolic syndrome [19,37]. However, the mechanisms of the effects of MUFA-rich oil or dietary-fiber-rich whole grains on menopausal disorders remain unclear. To address this, we explored the effects of tea seed oil and rice bran treatment in a multifactorial menopausal disorder model. OVX, d-galactose-injected, and chronically fructose-fed mice exhibit similar physiological changes to those that are incurred through aging [38,39,40]. In our study, old female mice underwent ovariectomy and after were treated with d-galactose and fructose for 8 weeks, after which they developed peripheral oxidative damage and insulin resistance. However, the mice aged 6–8 months may be considered too old to easily induce noticeable aging effects compared with a group of mice of the same age range without OVX and d-galactose treatment.

Studies showed that both polyphenols and dietary fiber may modulate gut microbiota, bringing health benefits [41,42]. A study also showed that rice bran contains both components, which regulate the gut microbiota and influence the production of SCFAs [43]. The potential bioactive compound in rice bran is γ-oryzanol; oral gavage of γ-oryzanol (100 mg/kg) in mice for three weeks can revert LPS-induced cognitive and memory impairments by promoting brain anti-inflammatory molecular responses [44]. In this study, 100 g of rice bran contained 550 mg of polyphenols, of which 11.9 mg was γ-oryzanol. The γ-oryzanol intake of mice was approximately 1 mg/kg. Thus, we hypothesized that the reduction in brain inflammatory responses might have been attributable to the γ-oryzanol in rice bran. However, this dosage remains insufficient to improve cognitive function in this multifactor-induced animal model.

Abnormal metabolism in the liver and altered gut microbiota jointly influence brain function through metaflammation that is induced by hepatic steatosis and harmful toxins produced in the gut, and this influence is evidence of the gut–liver–brain axis [45]. In the present study, linear regression revealed that the presence of specific microbiota, including *Tannerellaceae* and *Clostridia*, was associated with the levels of several brain and liver biomarkers. Burz et al. identified a higher relative abundance of *Tannerellaceae* in the feces of patients with nonalcoholic fatty liver disease (NAFLD) than in those from healthy controls, indicating that NAFLD might correlate positively with the abundance of *Tannerellaceae*, which secretes endotoxins in the gut and accelerates the progression of liver damage [46,47]. In the current study, the OSR and OTR groups exhibited significantly lower *Tannerellaceae* abundance than did the OS and OT groups. This lower abundance may be associated with improved liver function. *Clostridia* is one of the main microbiota that ferments insoluble dietary fiber to produce SCFAs, producing butyrate for energy and other uses [48]. In our study, *Clostridia* abundance was higher in the OSR and OTR groups than in the OS and OT groups, which may have caused greater butyrate production.

In our study, the mice fed a tea-seed-oil-based diet exhibited significantly greater accumulation of fat in the liver than did those fed different diets. Previous studies have reported that replacing dietary saturated-fatty-acid-rich palm oil with MUFA-rich olive oil in an apolipoprotein E^−/−^ mice model elevates the levels of adipose-differentiation-related proteins, which is strongly associated with the cellular TG content [49,50], and that for individuals with a MUFA-rich diet, the presence of fructose might enhance the accumulation of fat in the liver [51]. Fatty liver causes low-grade inflammation in the whole body and causes peripheral oxidative damage, which might increase the production of endotoxins and lead to aggravation of the proinflammatory response in the brain.

Although totally replacing dietary oil with tea seed oil can contribute to an imbalance in the gut–liver–brain axis, additional rice bran supplementation might alleviate the adverse effects of such a change. In our study, the ingestion of dietary-fiber-rich rice bran caused the relative abundance of *Clostridia* and the levels of SCFAs, especially butyrate, to increase. The delivery of butyrate-producing probiotics may regulate glucose and lipid metabolism in the liver by activating G protein-coupled receptor 41/43 or by inhibiting histone deacetylase, which would attenuate the progression of hepatic steatosis [52] and reduce the severity of metaflammation [15]. Consistently with previous studies, our data reveal that in mice fed tea seed oil and rice bran, the relative weight of the liver, the hepatic TG and TC content, AST and ALT activity, brain IL-1β and TNF-α levels, and 8-OHdG levels were significantly lower than those in mice fed other diets, indicating that rice bran supplementation has a protective effect against liver damage. SCFAs both reduce fat accumulation in the liver and directly or indirectly modulate the secretion of proinflammatory cytokines in the brain [53,54]. Our study demonstrated that in mice fed rice bran with tea seed oil, metaflammation and peripheral oxidative damage was reduced, and increased brain and liver SCFA levels enabled regulation of the gut–liver–brain axis, which led to reduced brain IL-1β and TNF-α levels.

The limitation of this study is that due to the high cost of 16S rRNA sequencing, we could only select and test five mice from each group out of a total of eight. Additionally, the OVX mice were already aged, and the feeding period may need to be longer. Consequently, no effects on spatial memory were observed in the water maze experiment.

## 5. Conclusions

Rice bran ameliorated the imbalance of the gut–liver–brain axis when administered with a tea-seed-oil–based diet by elevating the relative abundance of SCFA-producing *Clostridia* and reducing the relative abundance of endotoxin-producing *Tannerellaceae*. Rice bran supplementation inhibited the accumulation of hepatic fat, which reduced the severity of metaflammation and peripheral oxidative damage and led to decreased secretion of proinflammatory cytokines in the brain.

## Data Availability

The original contributions presented in the study are included in the article/Supplementary Materials, further inquiries can be directed to the corresponding author.

## Supplementary Materials

The following supporting information can be downloaded at: https://www.mdpi.com/article/10.3390/nu16172980/s1, Table S1: Composition of the experimental diets.
