# Supplementary material for: The Effects of Rice Bran on Neuroinflammation and Gut Microbiota in Ovariectomized Mice Fed a Drink with Fructose
Source: Nutrients. 2024 Sep 4;16(17):2980. doi: 10.3390/nu16172980 (PMC11397027; doi:10.3390/nu16172980)
Supplement: Supplementary file 1 [file nutrients-16-02980-s001.zip › nutrients-3181725-supplementary.pdf]

Table S1: Composition of experimental diets

	SY	SO	OS	OSR	OT	OTR
	g/kg	g/kg	g/kg	g/kg	g/kg	g/kg
Corn starch	405	405	405	393	405	393
Maltodextrin	155	155	155	155	155	155
Sucrose	100	100	100	89.9	100	89.9
Casein	140	140	140	121.5	140	121.5
L-cysteine	2	2	2	2	2	2
Soybean oil	100	100	100	90.3	-	-
Tea seed oil	-	-	-	-	100	90.3
Cellulose	50	50	50	15.4	50	15.4
Mineral mix (AIN-93M-MIX)	35	35	35	22.2	35	22.2
Vitamin mix (AIN-93M-MIX)	10	10	10	9	10	9
Choline bitartrate	3	3	3	3	3	3
tert-butylhydroquinone	0.008	0.008	0.008	0.008	0.008	0.008
Rice bran	-	-	-	100	-	100
Total (g)	1000.008	1000.008	1000.008	1001.308	1000.008	1001.308
kcal/g	4.108	4.108	4.108	4.103	4.108	4.103

SY: sham + young mice, SO: sham + old mice, OS: OVX mice + soybean oil diet, OSR: OVX mice + soybean oil with rice bran diet, OT: OVX mice + tea seed oil diet, OTR: OVX mice + tea seed oil with rice bran diet. Corn starch, maltodextrin, casein, cellulose, mineral mix, and vitamin mix were purchased from Hong Sheng Instruments (Taipei, Taiwan). L-Cysteine (Sigma-Aldrich, St. Louis, MO, USA), choline bitartrate (Sigma-Aldrich, St. Louis, MO, USA), and *tert*-butylhydroquinone (Sigma-Aldrich, St. Louis, MO, USA) were purchased from UNI-ONWARD (New Taipei, Taiwan). Sucrose, soybean oil, and tea seed oil were purchased from Taiwan Sugar Corporation (Tainan, Taiwan). Rice bran was provided by Professor Hitoshi Shirakawa, Tohoku University (Sendai, Japan).
